# Fibronectin-Expressing Mesenchymal Tumor Cells Promote Breast Cancer Metastasis

**DOI:** 10.3390/cancers12092553

**Published:** 2020-09-08

**Authors:** Brian H. Jun, Tianqi Guo, Sarah Libring, Monica K. Chanda, Juan Sebastian Paez, Aparna Shinde, Michael K. Wendt, Pavlos P. Vlachos, Luis Solorio

**Affiliations:** 1 School of Mechanical Engineering, Purdue University, West Lafayette, IN 47907, USA; bjun@purdue.edu; 2 School of Electrical and Computer Engineering, Purdue University, West Lafayette, IN 47907, USA; guo246@purdue.edu; 3 Weldon School of Biomedical Engineering, Purdue University, West Lafayette, IN 47907, USA; slibring@purdue.edu (S.L.); mchanda@purdue.edu (M.K.C.); 4 Department of Medicinal Chemistry and Molecular Pharmacology, Purdue University, West Lafayette, IN 47907, USA; jpaezpae@purdue.edu (J.S.P.); shinde.aparna014@gmail.com (A.S.); mwendt@purdue.edu (M.K.W.); 5 Purdue Center for Cancer Research, Purdue University, West Lafayette, IN 47907, USA

**Keywords:** fibronectin, epithelial mesenchymal transition, plasticity, breast cancer, extracellular matrix, premetastatic niche, metastasis, cell migration tracking, microfluidics

## Abstract

**Simple Summary:**

For cancer to metastasize, tumor cells must not only invade the local tissue but must also grow and proliferate once they arrive. Tumor cell heterogeneity, the presence of multiple types of cancer cells within a tumor, can increase cell proliferation and invasion through cooperative interactions, increasing the potential for metastasis. We recently found that pro-invasion cancer cells express the protein fibronectin and increase metastasis for pro-growth cancer cells. We investigated this interaction by analyzing these two cell types’ migration and survival, both alone and in co-cultures. We find that pro-invasion cells have a protective effect on pro-growth cells, which otherwise die after two days in nutrient-starved conditions. Further, we find that adding soluble fibronectin to pro-growth cells in culture was sufficient to improve survival. However, their survival was higher for co-culturing conditions. These studies highlight the importance of cancer cell heterogeneity and the role of fibronectin in metastasis.

**Abstract:**

Tumor metastasis is connected to epithelial-mesenchymal heterogeneity (EMH) and the extracellular matrix (ECM) within the tumor microenvironment. Mesenchymal-like fibronectin (FN) expressing tumor cells enhance metastasis within tumors that have EMH. However, the secondary tumors are primarily composed of the FN null population. Interestingly, during tumor cell dissemination, the invasive front has more mesenchymal-like characteristics, although the outgrowths of metastatic colonies consist of a more epithelial-like population of cells. We hypothesize that soluble FN provided by mesenchymal-like tumor cells plays a role in supporting the survival of the more epithelial-like tumor cells within the metastatic niche in a paracrine manner. Furthermore, due to a lower rate of proliferation, the mesenchymal-like tumor cells become a minority population within the metastatic niche. In this study, we utilized a multi-parametric cell-tracking algorithm and immunoblotting to evaluate the effect of EMH on the growth and invasion of an isogenic cell series within a 3D collagen network using a microfluidic platform. Using the MCF10A progression series, we demonstrated that co-culture with FN-expressing MCF10CA1h cells significantly enhanced the survival of the more epithelial MCF10CA1a cells, with a two-fold increase in the population after 5 days in co-culture, whereas the population of the MCF10CA1a cells began to decrease after 2.5 days when cultured alone (*p* < 0.001). However, co-culture did not significantly alter the rate of proliferation for the more mesenchymal MCF10CA1h cells. Epithelial tumor cells not only showed prolonged survival, but migrated significantly longer distances (350 µm compared with 150 µm, respectively, *p* < 0.01) and with greater velocity magnitude (4.5 µm/h compared with 2.1 µm/h, respectively, *p* < 0.001) under co-culture conditions and in response to exogenously administered FN. Genetic depletion of FN from the MCF10CA1h cells resulted in a loss of survival and migration capacity of the epithelial and mesenchymal populations. These data suggest that mesenchymal tumor cells may function to support the survival and outgrowth of more epithelial tumor cells within the metastatic niche and that inhibition of FN production may provide a valuable target for treating metastatic disease.

## 1. Introduction

Metastasis is the leading cause of cancer-related patient mortality [1,2,3,4]. Particularly for breast cancer, the five-year survival rate of patients is close to 99% if the disease remains localized, but decreases significantly if the disease progresses to other locations [5,6,7,8]. Cancer spreads throughout the body by a process known as metastasis, during which cancer cells escape from the primary tumor, disseminate into distal tissues, and proliferate to form a new secondary tumor [9]. Interestingly, the cells that have the highest capacity for invasion are often not capable of new tumor formation [10]. For new tumor formation, the cells that invade must also have the capacity to enter the growth cycle [11,12]. 

As a consequence, tumor heterogeneity can play an essential role in metastasis by allowing mixed populations of cells to invade and proliferate within distal tissues [10]. One source of heterogeneity occurs through epithelial-mesenchymal plasticity (EMP). During this process, polarized epithelial cells respond to microenvironmental stimuli and lose their apicobasal polarity, have a reduced proliferative capacity, develop increased resistance to drugs, and gain invasive properties [4,13,14]. Differential response of tumor cells to the microenvironmental stimuli can lead to the development of epithelial-mesenchymal heterogeneity (EMH) within a tumor [15]. The resultant phenotypic diversity within the tumor allows for unique paracrine signaling dynamics to develop between the different populations that are present in the system, which ultimately enhances the metastatic potential of the tumor [10].

Such paracrine signaling phenomena resemble wound healing or organ fibrosis events that involve the deposition of growth factors, hormones, and matrix-remodeling proteins to induce behavior in adjacent cells in the tissue [16]. Within tumors, the extracellular matrix protein, fibronectin (FN), is among the first to be upregulated in the developing tumor and is also observed in early metastatic lesions [17]. FN exists in both soluble globular and in-soluble fibrillar conformations. Our recent findings indicate that tumor cells in a stable mesenchymal phenotype are an abundant source of soluble FN, but the FN-producing tumor cells cannot complete the metastatic process by themselves. Instead, the FN-producing tumor cells enhance the metastasis of responder epithelial tumor cells in a paracrine manner [10,18]. 

To better understand the role of secreted FN within heterogeneous tumors during metastasis, we investigated the effects of co-culture on cancer cell migration and the survival of an MCF10A progression series by using a multi-parametric cell tracking algorithm and immunoblotting. Previous work with this progression series has shown that the metastatic potential of mosaic tumors was significantly enhanced relative to tumors inoculated using either homogeneous cell type [10]. However, it is not clear how the mesenchymal population enhanced the metastasis of the more epithelial population. Here, we observed that paracrine signaling between stably mesenchymal tumor cells plays a vital role in the survival of the more proliferative epithelial fraction. Furthermore, the soluble form of FN significantly contributes to increased survival. 

## 2. Results

### 2.1. Comparison of Measurements within Culture Groups

**Figure 1:**
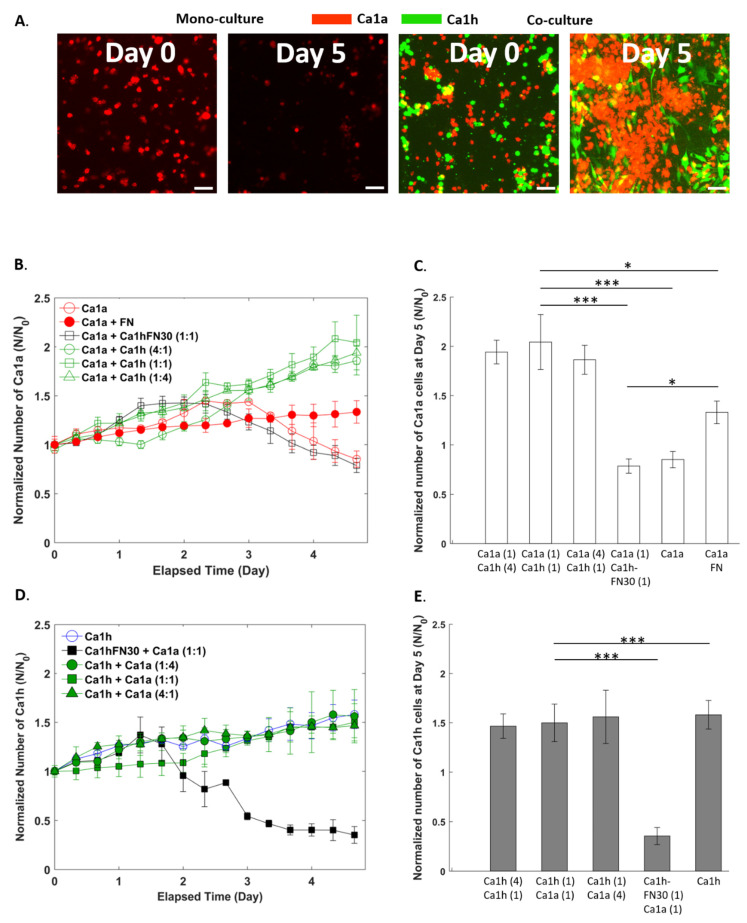
Proliferation of MCF10CA1a (Ca1a) and MCF10CA1h (Ca1h) cells in mono-culture and co-culture within the microfluidic chamber. (**A**) Wide-field 4× objective fluorescent microscopy images of the mono-cultured and co-cultured Ca1a cells. The scale bar shows 100 µm. (**B**) Normalized number of Ca1a cells across culture conditions and (**C**) at the terminal time point. (**D**) Normalized number of Ca1h cells across culture conditions and (**E**) at the terminal time point. *p*-values are denoted with asterisks (*) as follows: *: significant at *p* < 0.05, and ***: significant at *p* < 0.001.

**Figure 2:**
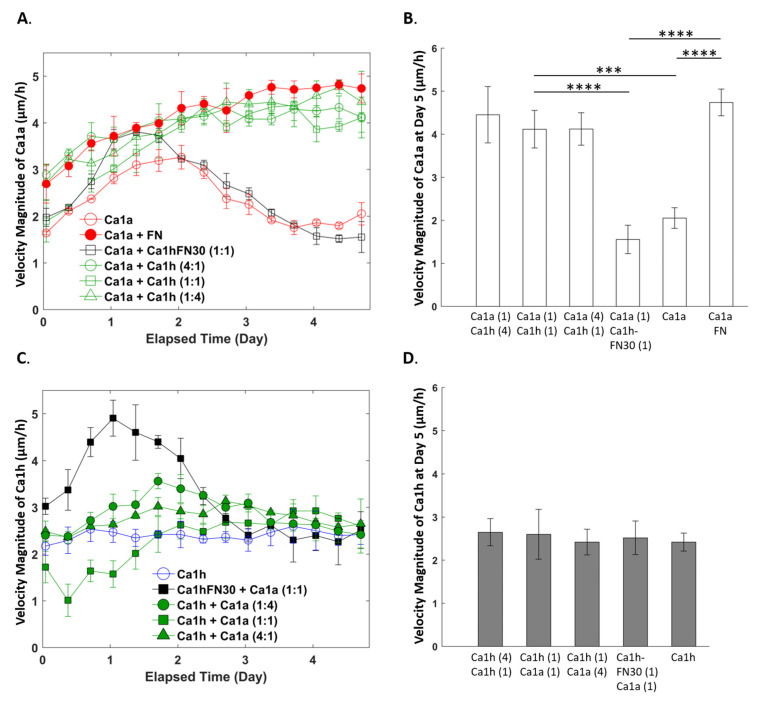
Velocity of Ca1a and Ca1h cells in mono-culture and co-culture within the microfluidic chamber. (**A**) Mean velocity magnitude of Ca1a and (**B**) at the terminal time point. (**C**) Mean velocity magnitude of Ca1h cells across culture conditions and (**D**) at the terminal time point. *p*-values are denoted with asterisks (*) as follows: ***: significant at *p* < 0.001, and ****: significant at *p* < 0.0001.

**Figure 3:**
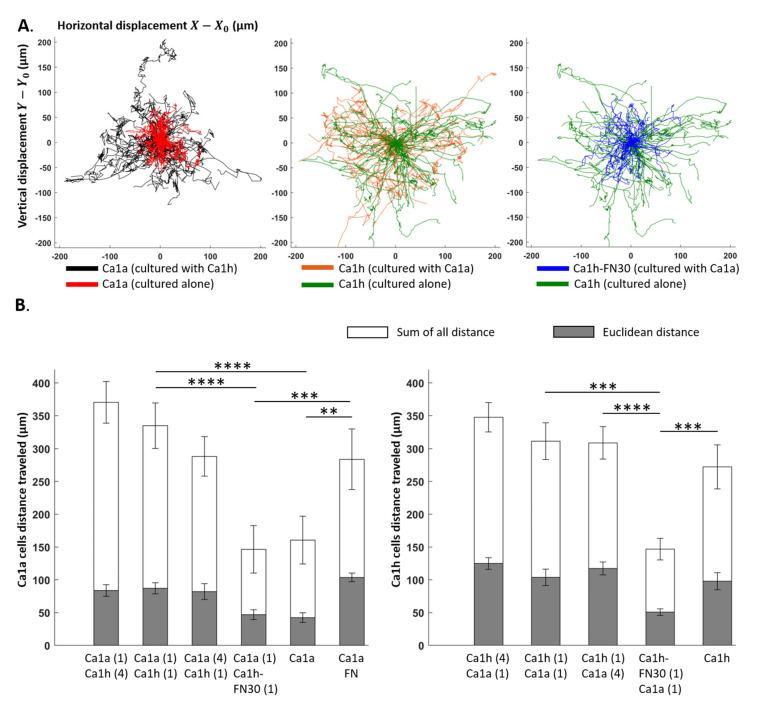
Trajectory of Ca1a and Ca1h cells in mono-culture and co-culture within the microfluidic chamber. (**A**) Tumor cell trajectory maps showing the full migration records of the representative 50 cells with the longest travel distance. (**B**) Mean Euclidean and the sum of all distances traveled quantified for Ca1a and Ca1h cells across the culture conditions. *p*-values are denoted with asterisks (*) as follows: **: significant at *p* < 0.01, ***: significant at *p* < 0.001, and ****: significant at *p* < 0.0001.

**Figure 4:**
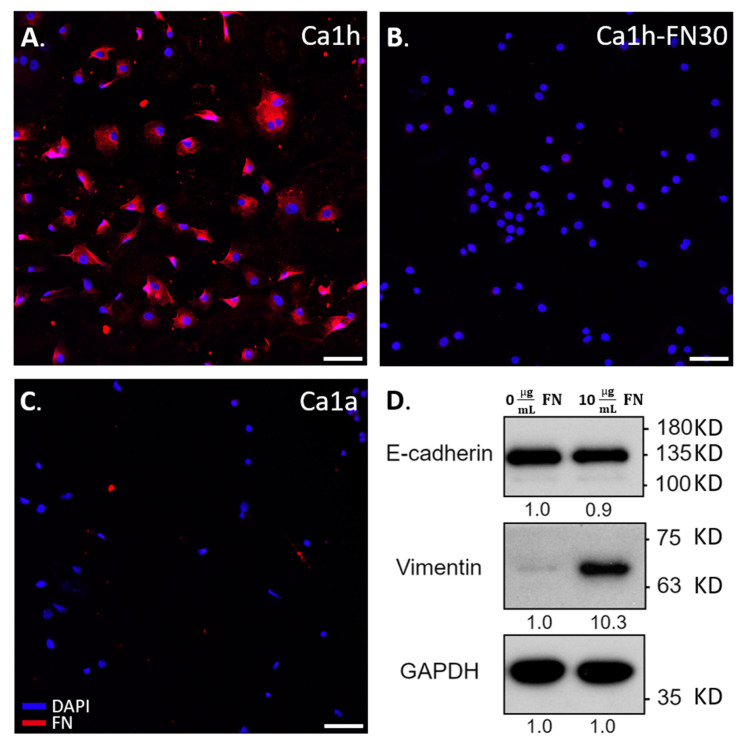
EMT markers in Ca1a cells upon fibronectin (FN) exposure within the well plate. The scale bar shows 100 µm. (**A**) Immunohistochemical staining for FN and DAPI on mono-cultured Ca1h cells, (**B**) Ca1h-FN30 cells, (**C**) Ca1a cells, (**D**) Immunoblotting with molecular weight markers and normalized intensity ratio, representing detection of EMT markers from Ca1a cells for FN content in their media.

**Figure 5:**
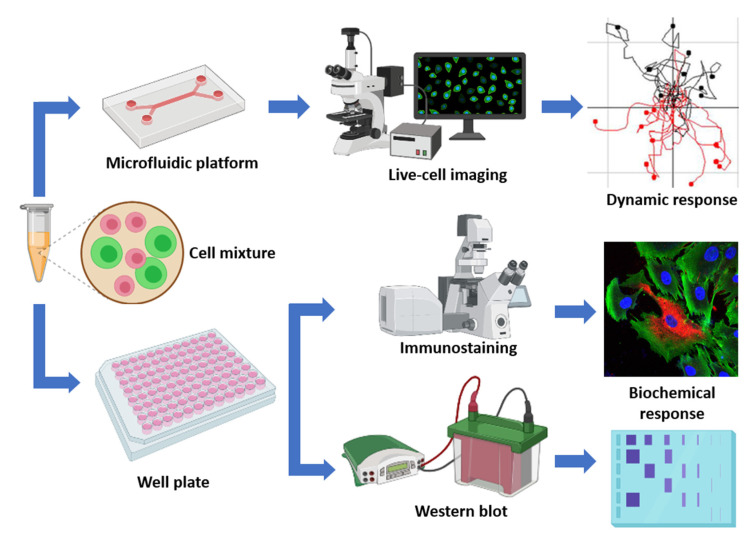
In vitro tumor microenvironment workflow allowing analysis of tumor cell growth and migration, immunohistochemical staining, and immunoblotting under varying degrees of co-culture conditions (Schematic created with BioRender.com).

**Figure 6:**
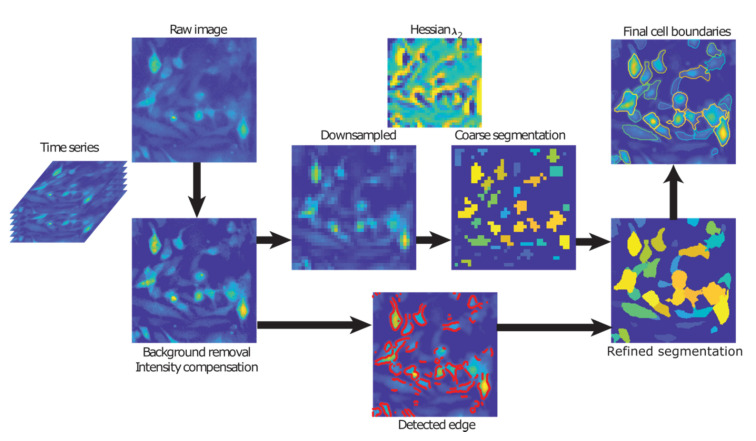
Image processing steps from raw image acquisition to segmentation of final cell boundaries.

**Figure 7:**
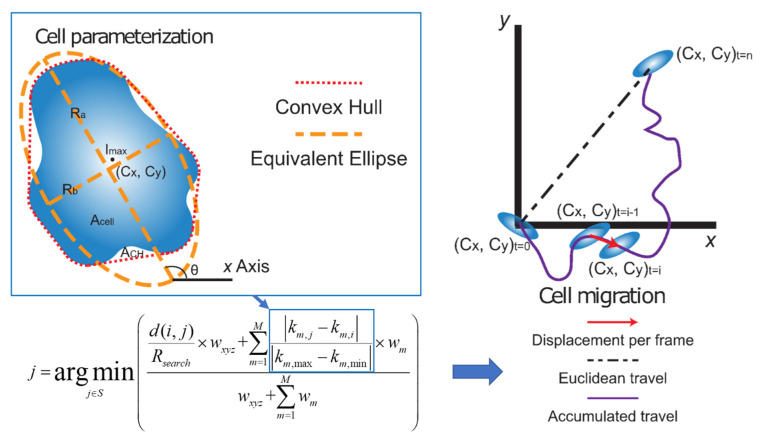
Cell parameterization and tracking representation where extracted cell parameters (*k*_1,2,3,∙∙∙,*M*_), together with spatial information (*x*, *y*, *z*) and associated weights (*w_xyz_* and *w_m_*), are used to find the most probable matching cells between image frames *i* and *j*. The pairing cell *j* is chosen as the one with the minimum weighted deviation of parameters from cell *i* in the first image.

Within the MCF10A progression series, the MCF10CA1a (Ca1a) and MCF10CA1h (Ca1h) lines have been established, which are epithelial- and mesenchymal-like, respectively. To investigate if changes in the rate of proliferation (Figure 1) or changes in invasion (Figure 2 and Figure 3) could be attributed to FN, we used the previously-developed Ca1h-FN30 cell line, in which FN was genetically depleted from Ca1h cells using shRNA [10]. Here, heterogenous samples were prepared using different mixture ratios of Ca1h and Ca1a cells, as well as a mixture of Ca1a and Ca1h-FN30 cells to test if the growth rate of Ca1a cells was dependent on the number of FN-expressing Ca1h cells. In addition, to determine if soluble FN was sufficient to produce the survival effects attributed by the Ca1h cells, we supplemented Ca1a mono-cultures with 10 µg/mL soluble FN. The 10 µg/mL is comparable to the level of FN previously quantified from conditioned media of 2D Ca1h cultures [17]. Overall, these conditions represent a primary tumor population expressing varying levels of FN. Subsequently, changes in epithelial-to-mesenchymal transition (EMT) markers in response to supplementation with soluble FN were evaluated, and included epithelial cadherin (E-cadherin) and vimentin as measured by immunoblotting (Figure 4 and Figure S1). Table S1 in the Supplementary summarizes the tested conditions. A detailed description of the cell lines, the microfluidic chamber, and corresponding analysis used in Figure 1, Figure 2, Figure 3 and Figure 4 to detect dynamic and biochemical responses upon exposing Ca1a cells to FN can be found in Section 4 (Figure 5, Figure 6 and Figure 7).

### 2.2. Effect of Co-Culture on Cell Proliferation 

A significant increase in the Ca1a population after 5 days was observed when co-cultured with Ca1h cells (Figure 1A). Figure 1B–E presents the growth rate of the cells that was calculated from the cell counts from each segmented image normalized by the initial cell count (N/N_0_). A detailed cell counting procedure can be found in Section 4 along with Figure 6 and Figure S2. Figure 1B shows that the mono-cultured Ca1a cells showed a 38% increase in the cell population by 2.5 days without the presence of Ca1h cells or exogeneous-FN. After 2.5 days, the cell number decreased for the duration of the study. Such a trend is expected due to the limited amount of fetal bovine serum (FBS) and nutrients available within the system. 

Interestingly, under 1:1 co-culture with FN-expressing Ca1h cells, we observed a continued increase in the Ca1a population throughout the 5-day study. As a result, the Ca1a population was doubled by the end of the culture, with no decrease in cell number occurring within the observed time domain. Next, we varied the ratio of Ca1a to Ca1h cells to determine if the heterogeneously cultured Ca1a cells with Ca1h cells would affect the growth kinetics and survival of the Ca1a cells. Between 0 and 3 days, the 1:4 ratio (which is the lowest level of Ca1h cells) had the slowest growth rate among the three different co-culture conditions. Nevertheless, the growth rate continued to increase, and at the end of the culture period, no statistical significance in the normalized number of cells was observed across the different ratios. After 5 days in culture, the number of co-cultured Ca1a cells had doubled, while the mono-cultured Ca1a cells were lower than what was observed after the initial seeding (Figure 1C). The statistical analysis is described in Section 4. 

To determine if FN altered the growth kinetics, we co-cultured the Ca1a cells with Ca1h-FN30 cells. A significant decrease in the normalized number of Ca1a cells was observed relative to the co-culture with the Ca1h cells. However, no significant differences were observed in the cell number dynamics of the Ca1a cells when cultured alone or with the Ca1h-FN30. To account for the additional signaling factors produced by Ca1h cells, we then cultured Ca1a cells with exogenously supplemented FN. Ca1a cells cultured with exogenously supplemented FN reached a 34% population increase at the end, which was lower than the co-culture conditions with the Ca1h cells. However, when FN was supplemented, the Ca1a cell population did not have a decrease in cell number, as was observed between 1.5 and 2.5 days with the Ca1a mono-culture and with the Ca1a:Ca1h-FN30 co-culture. After 5 days in culture with exogenous FN, the Ca1a population was 58% greater than the Ca1a control (Figure 1C). 

In contrast, the growth rate of Ca1h cells was not affected significantly by the co-culture conditions and steady proliferation was observed throughout the 5-day culture (Figure 1D). The mono-cultured and co-cultured Ca1h cells reached a population increase between 47% and 56% after 5 days (Figure 1E). The only substantial changes occurred upon genetic depletion of FN (Figure 1D,E). After 1.2 days, the Ca1h-FN30 cell numbers began to decrease and reached a 65% reduction after 5 days, whereas the Ca1h cell proliferation was not affected by the co-culture conditions. Within the Ca1a:Ca1h-FN30 co-culture condition, no survival advantage was observed for either cell line. Overall, after 5 days, the number of Ca1h cells observed in all cultures was lower than the number of Ca1a cells co-cultured with Ca1h cells (Figure 1E). 

### 2.3. Effect of Co-Culture on Cell Migration Velocity

Figure 2A shows the Ca1a cells time-varying mean velocity magnitude across different culture conditions. The mono-culture of Ca1a cells (control) reached a maximum velocity magnitude of 3.3 µm/h after 2 days in culture. Upon reaching maximum velocity, the velocity decreased for the next 2 days, until it reached a minimum of 1.8 µm/h. This velocity did not significantly change for the remainder of the study. Under co-culture conditions, the velocity of the Ca1a cells increased through the first 3 days in culture, reaching a peak velocity of 4.8 µm/h. No significant changes in velocity occurred until the end of the culture. Ca1a cells cultured with Ca1h-FN30 cells reached a maximum velocity of 3.9 µm/h after just 1 day in culture. Afterward, the velocity decreased through the remaining 4 days in culture to a minimum of 1.5 µm/h, and no significant changes were observed. No statistically significant difference in the velocity of Ca1a cells was observed among the three co-culture conditions that contained FN expressing cells (Figure 2B). The Ca1a cells in mono-culture supplemented with 10 µg/mL of FN showed a similar velocity profile and magnitude to those co-cultured with Ca1h cells.

The mono-cultured and co-cultured Ca1h population had a lower velocity magnitude than the Ca1a cells co-cultured with Ca1h cells throughout the 5-day culture (Figure 2C). While co-culture appeared to affect the velocity of the Ca1h cells within the first 3 days, no clear pattern was observed in response to the Ca1a:Ca1h ratio with variation between 1 and 3.6 µm/h for the first 3 days. After 3 days, no significant difference was observed in the Ca1h velocity regardless of the co-culture conditions (Figure 2D). The velocity profile for the Ca1h-FN30 cells in Figure 2C shows a trend that is similar to their epithelial counterparts from Figure 2A. The Ca1h-FN30 cells reached a maximum velocity magnitude in 1 day, after which the velocity magnitude decreased until the end of the culture. Interestingly, the observed velocity profile of the Ca1h-FN30 followed what was observed with the Ca1a mono-culture.

### 2.4. Effect of Co-Culture on Trajectory

Figure 3A shows a representative trajectory map of the Ca1a and Ca1h cells across different culture conditions. The motility of each tumor cell phenotype was compared by quantifying the Euclidean distance, accumulated traveled distance, forward migration index, and directness (Figure 3B and Table S2). These parameters were quantified by choosing 100 segmented cells with the longest tracks within each sample. This method was applied in order to minimize sample to sample variation in terms of the number of segmented cells, to discount tracks that are no longer than 10 frames which can significantly bias mean values, and to eliminate any effects that dead cells may have on the measurement output. Each culture condition was performed in triplicate, and each of the averaged quantities consists of 300 individual cell tracks. 

As anticipated, the sum of all distances was higher than the Euclidean distance for all conditions studied (Figure 3B). Both the Euclidean distance and the sum of all distances traveled for the Ca1a cells was significantly higher (*p* < 0.001) in all three co-culture conditions with FN-expressing Ca1h cells than in mono-culture. Similar to the cell proliferation and velocity studies (Figure 1 and Figure 2), the ratio of the Ca1h:Ca1a cells had no statistically significant effect on the Euclidean distance or sum of all distances traveled. Ca1a cells in mono-culture with exogenously supplemented FN had a significantly higher sum of all distances and Euclidean distance travel path than the Ca1a mono-culture without FN. However, no statistical differences were observed between the distances traveled by Ca1a cells supplemented with FN and those co-cultured with FN-expressing Ca1h cells. The same was observed in the distances traveled between mono-culture Ca1a cells and those co-cultured with Ca1h-FN30 cells (Figure 3B). 

Likewise, the observation of the Euclidean distance or the sum of all distances traveled for the Ca1h cells within the co-culture conditions showed no statistical differences. Additionally, the Ca1h-FN30 had a significantly lower Euclidean distance and the sum of all distances traveled than all the other Ca1h groups (*p* < 0.001). 

In addition to the mean traveled distance values presented in Figure 3B, several chemotactic parameters were measured as well (Table S2). The forward migration index (FMI) represents the directed chemotactic cell migration towards the chemoattractant source. Subsequently, the FMI^׀׀^ with a value of -1 would signify that the cells are migrating towards the chemoattractant along a straight path. In this study, no statistically significant chemotactic behavior was observed with respect to all conditions, as the mean FMI values were close to 0. A potential reason for this could be that the chemoattractant source concentration was not high enough for the cells to respond more actively. 

The mean directness index (D) did not show any statistically significant differences across all culture conditions. The mean value measured was 0.42, while the maximum directness possible would be 1 (which would represent a perfectly straight trajectory of cells from start to finish). While higher chemoattractant source concentrations may allow observation of the directed movement of specific phenotypes, it can also saturate the nutrient level in the ECM. This causes the uninterrupted growth of these cells across the test conditions, which must be avoided. 

### 2.5. Immunohistochemical Staining and Immunoblotting

Immunohistochemical staining of Ca1h and Ca1a cells indicated that FN was only detected in the mesenchymal Ca1h fraction (Figure 4), verifying previous immunoblot results [10,15]. As expected, the Ca1h-FN30 did not express FN [10]. Due to the enhanced motility and survival of the Ca1a cells in the presence of soluble FN, we assessed common markers for EMT, E-cadherin, and vimentin (Figure 4 and Figure S1). Whole-cell lysates were collected from Ca1a cells after 72 h in culture with or without soluble FN. Ca1a cells in both conditions retained a majority of their E-cadherin expression. However, vimentin expression increased, indicating that the addition of 10 µg/mL of soluble FN potentially induced a partial EMT response. 

## 3. Discussion

Recent studies by our group indicated that EMH could significantly contribute to the metastatic cascade. To better delineate the effects that phenotypic heterogeneity has on tumor progression, we employed a cell tracking algorithm to evaluate the cellular dynamics throughout a prolonged 3D culture. Our cell tracking algorithm uses a multi-parametric approach to identify and track cells over time and space accurately [19,20]. Additionally, we used immunoblotting to measure changes in EMT markers within Ca1a cells upon exposure to FN. 

Using the isogenic Ca1a and Ca1h cell series, we were able to evaluate the effect that a stable mesenchymal cell population would have on a more epithelial counterpart. The data presented here clearly demonstrate that FN-expressing Ca1h cells increased the survival, velocity magnitude, and migrated distance of Ca1a cells by a factor of two within an in vitro 3D microfluidic culture system (Figure 1, Figure 2 and Figure 3). These features were lost when Ca1a cells were co-cultured with Ca1h cells that are depleted of FN. However, these trends were partially recovered with the introduction of exogenous FN to the mono-culture of Ca1a cells. While soluble FN did not enable the more significant proliferation rate seen by the Ca1a cells when co-cultured with Ca1h cells, exogenous FN enabled the survival of the existing Ca1a cells with only moderate growth in total numbers over the culture period (Figure 1B,C), indicating that other factors contribute to the observed increase in proliferation. 

Beyond FN, it has been reported that Ca1h cells are better equipped than Ca1a cells to process lactate [21]. Furthermore, lactate has been demonstrated to play a role in inhibiting tumor cell proliferation [22]. In this way, the Ca1a cells alone likely could not overcome the metabolically stressed environment created within the microfluidic channel (Figure 1B). Additionally, when the Ca1a cells were cultured with soluble FN, an increase in vimentin was observed, which may indicate that the cells were undergoing a partial EMT (Figure 4D). Previous literature has demonstrated that a post-EMT phenotype has increased survival and lower proliferation than the epithelial tumor fraction [23,24]. As such, various pathways are initiated by the FN-integrin signaling axis. In particular, β1 integrin has been shown to activate STAT3 via a FAK and Jak2 dependent pathway [11,25]. Therefore, studies to elucidate the role of these signaling molecules in driving the survival of the Ca1a cells are warranted. Beyond survival, we find that, when epithelial cells are co-cultured with FN-expressing mesenchymal cells or soluble FN, they are able to travel very similar distances through the collagen microenvironment as the Ca1h cells (Figure 2 and Figure 3). Indeed, even when Ca1a cells are cultured without FN (Ca1h-FN30 co-culture or Ca1a mono-culture), the velocity magnitude is similar to the conditions with FN until approximately day 1.5–2, which is about 1 day before an overall reduction in cell number begins (Figure 1B). However, the exact mechanism of Ca1a migration through collagen is not known. It has been demonstrated that matrix metallopeptidase (MMP) production by MDA-MB-231 cells plays a significant role in enhancing cell migration through 3D collagen gels [26]. Therefore, it is likely that a post-EMT phenotype of the Ca1a cells may lead to enhanced production of MMPs. This study further validates the importance of phenotypic heterogeneity in the early stages of metastasis. Identifying the specific role of tumor heterogeneity and FN in local invasion is one aspect that should be addressed in future work. 

There exist several limitations in the present study. First, the cell motions were restrained to 2D space since the microfluidic chamber was designed to image cells reacting to a 1D chemotaxis gradient (Figure S3). In practice, the tumor cells in vivo exhibit more complex 3D motions. Second, the in vitro microfluidic tumor microenvironment system used in this work allows replication and study of primary tumor growth, but not secondary tumor growth via invasion. Lastly, detailed molecular-scale analysis evaluating the signaling mechanism between Ca1a cells and FN are required to advance our biological understanding of how tumor-promoting functions of FN and ECM-induced changes affect Ca1a cells. We expect that our analysis and principles can be applied to advanced measurements for interrogating metastatic progression. 

## 4. Materials and Methods 

### 4.1. Cell Culture

Isogenic MCF10CA1aand MCF10CA1h cells (Fred Miller, Wayne State University, Detroit, MI, USA), [10] were cultured in Dulbecco’s Modified Eagle’s Medium (DMEM; Gibco) containing 10% fetal bovine serum (FBS; Gibco), 1% penicillin/streptomycin (Gibco), and 1% Non-Essential Amino Acid (NEAA; Corning). As previously described, lentiviral transduction was used to stably express EGFP or dTomato [10]. The depletion of FN from Ca1h cells (Ca1h-FN30) was achieved through lentiviral-mediated transduction of TRCN0000064830, or a nontargeting scrambled control shRNA (Ca1h). Stable expression was selected with puromycin [10]. Cell cultures were maintained in a 5% CO_2_ atmosphere at 37 °C in an incubator. The Ca1a and Ca1h cells are epithelial and mesenchymal, respectively. Previous work has shown that the metastatic potential of mosaic tumors that consisted of a 1:1 mixture of Ca1a and Ca1h cells were significantly enhanced relative to tumors inoculated using Ca1a or Ca1h alone [10]. 

### 4.2. Microfluidic 3D Hydrogel Assays

Fluorescently labeled Ca1a (dTomato) and Ca1h (GFP) cells were used to track cell growth and migration within 3D collagen hydrogels. To create our cell-laden gels, we first concentrated 3 million cells into 100 µL of DMEM. Next, the cell solution was mixed with 8 mg/mL rat tail type I collagen solution (Corning), 10X PBS, and neutralized with sodium hydroxide for a final collagen concentration of 2.0 mg/mL, 1X PBS, a pH of 7.4, and a cell concentration of 3 million cells/mL. The cell-hydrogel mixture was then loaded into the microfluidic chambers (µ-Slide Chemotaxis; ibidi) by pipetting, and then incubated in a 5% CO_2_ atmosphere at 37 °C for 30 min to polymerize the collagen solution. Afterward, 60 µL DMEM was added to each of the two media reservoirs with 5% FBS on only one side of the reservoir to create a diffusion mediated chemoattractant gradient across the cell-loaded observation area. Finally, the microfluidic chambers were placed in an incubated stage for live-cell fluorescent microscopy imaging. The elapsed time between the loading of the cell-hydrogel mixture and the start of the imaging was approximately 2 h. The overall experimental workflow is shown in Figure 5. Additionally, a detailed description on the microfluidic chamber can be found in Figure S3 [27,28,29,30,31,32]. 

### 4.3. Time-lapse Imaging

Tumor cell migration was recorded using time-lapse imaging on a wide-field fluorescence inverted microscope with a motorized stage (DMI 6000 B, Leica Microsystems, Wetzlar, Germany), 1280 × 1024 CCD camera (Retiga 1300C, Teledyne Photometrics, Tucson, AZ, USA) and 4× magnification (1.34 µm/pix). Images were acquired at 1 frame per 30 min for 5 days (240 images). A mercury lamp was used for fluorescence illumination, with a fluorescence filter cube for imaging EGFP and dTomato labeled tumor cells. At each time point, multi-channel fluorescence images were acquired for each sample covering the observation area. Each image sequence was acquired with the focal plane in the center of the observation channel. Image acquisition was controlled through Leica Las X Life Science software. 

### 4.4. Cell Segmentation

Figure 6 illustrates the image processing steps for segmenting individual cells. Background subtraction was first applied to each frame in the time series to deal with low contrast ratios (CR) and compensate for uneven spatial illumination levels. A frame-wise linear intensity adjustment was applied, such that 1% of the total pixels were saturated, accounting for temporal fluorescence decay due to photobleaching. The images were down-sampled by a factor of 5 such that each pixel in the down-sampled image corresponded to the averaged intensity level in its 5 × 5 neighborhood from the original image, in order to avoid over-segmentation of the cells due to intracellular signal heterogeneity. A local Hessian matrix of the intensity was calculated for each pixel, and the cells (resembling bright tubes or blobs) were marked by negative λ_2_ values in the Hessian eigenmaps [19]. A dynamic erosion procedure with an adaptive threshold was used to identify each intensity peak of all cells that were analyzed. Subsequently, a dilation procedure was used to expand the boundaries from the identified peaks until it captures the course boundary of each cell. Finally, the coarse segmentation was mapped back to the original resolution and refined. The refining expansion stopped either when the pixel intensity fell below 25% of the peak intensity within the cell, or when it met the edges detected by a Canny filter [33]. 

### 4.5. Cell Parameterization

Our algorithm identifies the most probable correspondence between cells by taking into consideration the characteristics of each cell (brightness, morphology, etc.) in addition to the classic nearest-neighbor criterion as tracking parameters (Figure S2) [19,20,34,35]. Hence, cells were parameterized based on their refined segmentation and the equivalent ellipse, as shown in Figure 7.

From the equivalent ellipse the following parameters were used to identify the cell: the aspect ratio (*AR*, Equation (1)), solidity (*S*, Equation (2)), together with the equivalent radius (*R*, Equation (3)), orientation (θ) with respect to the positive x-direction, peak intensity (I_max_), and the intensity-weighted-centroid (*C_x_*, *C_y_*). The calculations for parameters are described below [36] and illustrated in Figure 7:

(1) $$AR=\frac{R_{b}}{R_{a}}$$

(2) $$S=\frac{A_{cell}}{A_{CH}}$$

(3) $$R=\sqrt{\frac{A_{cell}}{\pi }}$$

Each cell was then represented in the higher dimensional space by its feature vector (*C_x_*, *C_y_*, I_max_, *R*, θ, *S*, *AR*). Cell correspondence between frames was then determined by multi-parametric tracking to form cell migration tracks [19,20]. 

### 4.6. Quantifying Cell Growth and Motility

The growth rate of the cells was calculated from the cell counts from each segmented image normalized by the initial cell count (Equation (4)).

(4) $$\frac{N_{t=i}}{N_{t=0}}$$

Tracks obtained for each cell were then used to quantify cell migration. The displacement per frame (velocity) of each cell was obtained by taking the difference of the centroid points between adjacent frames (Equation (5)).

(5) $${\left(u,v\right)}_{t=i}={\left(C_{x},C_{y}\right)}_{t=i}-{\left(C_{x},C_{y}\right)}_{t=i-1}$$

The magnitude of which was used to construct the total velocity histograms for all cells over the entire time sequence. Then two total travel distances were calculated to describe the migration process (Equation (6)) [37,38].

(6) $$\{\begin{array}{c} d_{euc}=\sqrt{{\left(\overset{n}{\underset{t=1}{\sum }}u_{t}\right)}^{2}+{\left(\overset{n}{\underset{t=1}{\sum }}v_{t}\right)}^{2}}\\ d_{acc}=\overset{n}{\underset{t=1}{\sum }}\sqrt{u_{t}^{2}+u_{t}^{2}} \end{array}$$

The first Euclidean distance *d_euc_*, was calculated, which measures how far the cells traveled away from the starting point (*t* = 0) and endpoint (*t* = *n*) for each cell track. The second distance measured, *d_acc_* represents the total accumulated travel, which is the total migration distance that was tracked for each cell over time, which represents the motile nature of cells.

These two variables are used to analyze kinematic quantities of the migration process, such as the forward migration index and directness [39]. The forward migration index (FMI) was used to measure the effect of chemotaxis on cell migration and represents the efficiency of the forward migration of cells. Here, we define the x-axis as parallel to the chemotaxis gradient (׀׀, Equation (7)) and the y-axis as perpendicular to the chemotaxis gradient (⊥, Equation (8)). The larger the FMI, the stronger the chemotactic effect is on the x-axis or y-axis.

(7) $$FMI^{\mathrm{II}}=\frac{1}{n}\overset{n}{\underset{i=1}{\sum }}\frac{x_{i,end}}{d_{i,acc}}$$

(8) $$FMI^{⊥}=\frac{1}{n}\overset{n}{\underset{i=1}{\sum }}\frac{y_{i,end}}{d_{i,acc}}$$

The directness (Equation (9)) is a measure of the straightness of the cell trajectories, which indicates varying degrees of cell polarity across the different cell phenotypes [40]. It is calculated by comparing the Euclidean distance ($d_{euc}$) and the accumulated distance ($d_{acc}$) between the starting point and the endpoint of a migrating cell. Directness values are always positive. A directness of *D* = 1 equals a straight-line migration from the start to the endpoint.

(9) $$D=\frac{1}{n}\overset{n}{\underset{i=1}{\sum }}\frac{d_{i,euc}}{d_{i,acc}}$$

### 4.7. Immunohistochemical Staining and Immunoblotting

For immunoblotting, Ca1a cells were cultured in 6-well tissue culture-treated plates for three days in serum-free DMEM with 1% penicillin/streptomycin with or without 10 µg/mL FN, changing media as needed. After 72 h, cells were rinsed with 1X Phosphate-Buffered Saline (PBS; Gibco), lysed with 150 µL of RIPA buffer (Thermo Fisher Scientific, Waltham, MA, USA), shaken on ice for 20 min, and stored at −20 °C. The lysates were separated by reducing SDS-PAGE and probed for E-cadherin and vimentin (BD Biosciences, San Jose, CA, USA). For immunofluorescence, cells were fixed with 4% paraformaldehyde, permeabilized in 0.1% Triton-X 100, and processed using a polyclonal FN antibody (Invitrogen, Carlsbad, CA, USA), and DAPI for nuclear visualization. 

### 4.8. Statistics

All statistical analyses were performed using JMP software (SAS, Cary, NC, USA). A one-way ANOVA was applied to data from each characterization method to identify statistically significant interactions with co-culture parameters. Tukey’s HSD means comparison was used to determine the degree of significance (*p*-value) of interactions. In all subsequent figures *p*-values are denoted with asterisks (*) as follows: *: significant at *p* < 0.05, **: significant at *p* < 0.01, ***: significant at *p* < 0.001, and ****: significant at *p* < 0.0001. To represent the statistical significance clearly, comparison was done only between the 1:1 ratio co-culture conditions and mono-culture conditions, as we found no statistical significance across the different ratios of Ca1a to Ca1h co-culture groups with respect to normalized cell numbers, velocity magnitude, and distance traveled at day 5.

## 5. Conclusions

We presented measurements that evaluate the role of FN on the metastatic progression of breast cancer through assessing varying degrees of EMH in vitro. The data presented here demonstrate that both FN-expressing Ca1h cells and exogeneous FN facilitated the survival of more epithelial tumor cells within the in vitro microfluidic tumor microenvironment. This study evaluates the cellular dynamics of epithelial and mesenchymal populations that may play a role in the enhanced metastasis previously observed in vivo [10], and highlights the importance of phenotypic heterogeneity in the early stages of metastasis. Future investigation will emphasize targeting FN production and signaling in Ca1h and Ca1a cells, respectively, as a means of preventing tumor metastasis. 

## Supplementary Materials

The following are available online at https://www.mdpi.com/2072-6694/12/9/2553/s1, Table S1, Test conditions to evaluate the metastasis of Ca1a cells, Table S2, Summary of cell tracking parameters, Figure S1, Uncropped immunoblotting of the Ca1a cells, with molecular weight markers and densitometry readings, Figure S2, Number of cells counted from the published cell migration images using our cell tracking algorithm, Mosaic ImageJ plugin, and manual counting, and Figure S3, Ca1a and Ca1h cells in mono-culture and co-culture within µ-Slide Chemotaxis for measuring proliferation, velocity, and trajectory.
