# Supplementary material for: Fibronectin-Expressing Mesenchymal Tumor Cells Promote Breast Cancer Metastasis
Source: Cancers (Basel). 2020 Sep 8;12(9):2553. doi: 10.3390/cancers12092553 (PMC7565075; doi:10.3390/cancers12092553)
Supplement: Supplementary file 1 [file cancers-12-02553-s001.pdf]

cancers

Supplementary materials: Fibronectin-Expressing Mesenchymal Tumor Cells Promote Breast Cancer Metastasis

Brian H. Jun, Tianqi Guo, Sarah Libring, Monica K. Chanda, Juan Sebastian Paez, Aparna Shinde, Michael K. Wendt, Pavlos P. Vlachos and Luis Solorio

Table S1. Test conditions to evaluate the metastasis of Ca1a cells.

Expected Changes to Ca1a Cells upon FN Exposure		Measurement Approach	Test Samples with Varying Degrees of FN Exposure	
Dynamic responses	**↑ Cell proliferation**<br>**↑ Migration velocity**<br>**↑ Migration distance**	Cell segmentation and motility tracking analysis	Heterogeneous tumor	Ca1a + Ca1h (4:1)<br>Ca1a + Ca1h (1:1)<br>Ca1a + Ca1h (1:4)<br>Ca1a + Ca1h-FN30 (1:1)
			Homogeneous tumor	Ca1a (control)<br>Ca1h (control)<br>Ca1a + soluble FN
Biochemical responses	↓ E-cadherin<br>↑ Vimentin	Western blot analysis	Homogeneous tumor	Ca1a (control)<br>Ca1a + soluble FN

Table S2. Summary of cell tracking parameters.

Phenotype	Culture	FMI$^{\parallel}$	FMI$^{\perp}$	D	d_{euc} (μm)	d_{acc} (μm)
Ca1a	Homogeneous	-0.03±0.03	0.03±0.01	0.40±0.02	103.7±6.53	283.70±46.00
	Homogeneous + FN	0.00±0.02	-0.04±0.01	0.45±0.05	42.38±7.31	160.56±36.39
	Heterogeneous (Ca1h_FN30 to Ca1a ratio 1:1)	0.02±0.04	-0.02±0.07	0.43±0.03	46.83±7.41	146.48±36.22
	Heterogeneous (Ca1h to Ca1a ratio 2:8)	-0.01±0.02	0.02±0.02	0.38±0.01	82.03±12.10	288.22±30.03
	Heterogeneous (Ca1h to Ca1a ratio 1:1)	-0.02±0.05	-0.05±0.09	0.38±0.03	87.05±8.39	334.90±34.58
	Heterogeneous (Ca1h to Ca1a ratio 8:2)	0.00±0.03	0.00±0.02	0.31±0.02	83.81±8.82	370.41±31.83
Ca1h	Homogeneous	0.00±0.03	0.03±0.05	0.45±0.05	97.82±13.01	272.27±33.58
	Heterogeneous (Ca1h_FN30 to Ca1a ratio 1:1)	0.00±0.06	-0.03±0.09	0.51±0.02	50.67±5.09	146.80±16.52
	Heterogeneous (Ca1h to Ca1a ratio 2:8)	0.00±0.04	-0.08±0.06	0.42±0.04	117.37±9.85	308.77±24.62
	Heterogeneous (Ca1h to Ca1a ratio 1:1)	0.00±0.05	-0.02±0.08	0.43±0.06	103.78±12.55	311.37±27.90

Heterogeneous (Ca1h to Ca1a ratio 8:2)	0.00±0.04	-0.02±0.08	0.46±0.02	124.92±8.96	347.70±22.40

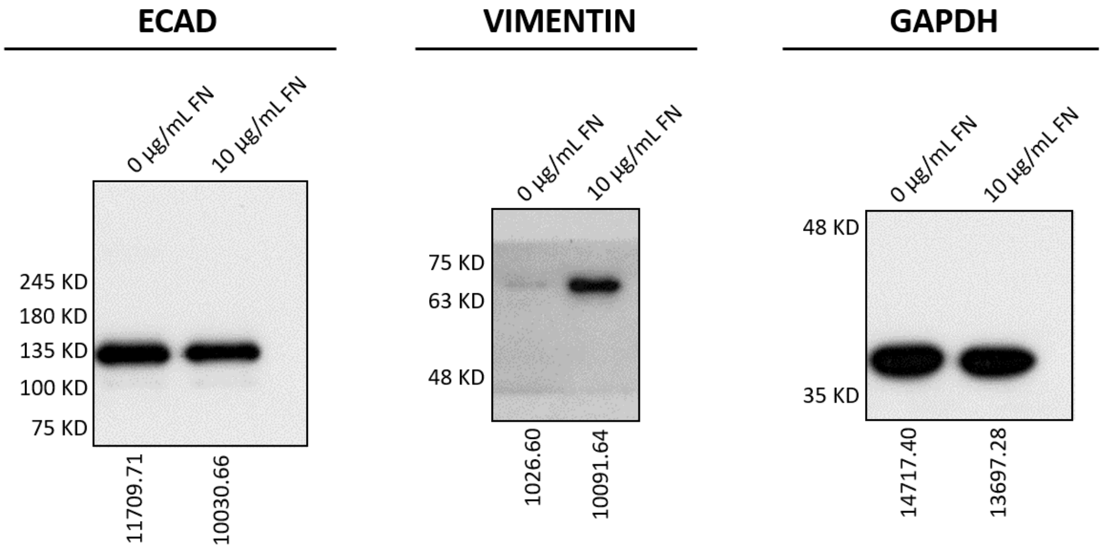

Figure S1. Uncropped immunoblotting of the Ca1a cells, with molecular weight markers and densitometry readings.

Algorithm performance on a benchmark data set

To benchmark the performance of the multiparametric cell tracking algorithm, we evaluated our output using a test case (PhC-C2DL-PSC, http://celltrackingchallenge.net/) from the cell tracking challenge. The test case was selected due to the similarity of the image series with our experimental data in terms of cell shape, migration behavior, and cell concentration [34]. The results were uploaded, verified, and published by the test organizers (2019-09-02 results), and the scores for cell detection and tracking performances were 0.94 and 0.93 (out of 1), respectively. The detailed evaluation methodology can be found from the website. Our results were compared against machine-learning based algorithms and manual tracking.

Additionally, comparative analysis was performed between the cell tracker plugin (Mosaic) available in Image J [35] and our multi-parameter algorithm using the aforementioned test case. The cell counter plugin was able to detect an accurate number of cells until the 100th frame, which had 131 cells present within the image. However, as the number of cells increased, on the 300th frame, only 242 cells were detected out of 709 cells, with the manual count used as the ground truth (Figure S1). Despite the rapid proliferation and change in the cell shape across the image series, our method was able to accurately detect all the visible cells even when the cells were closely located next to each other.

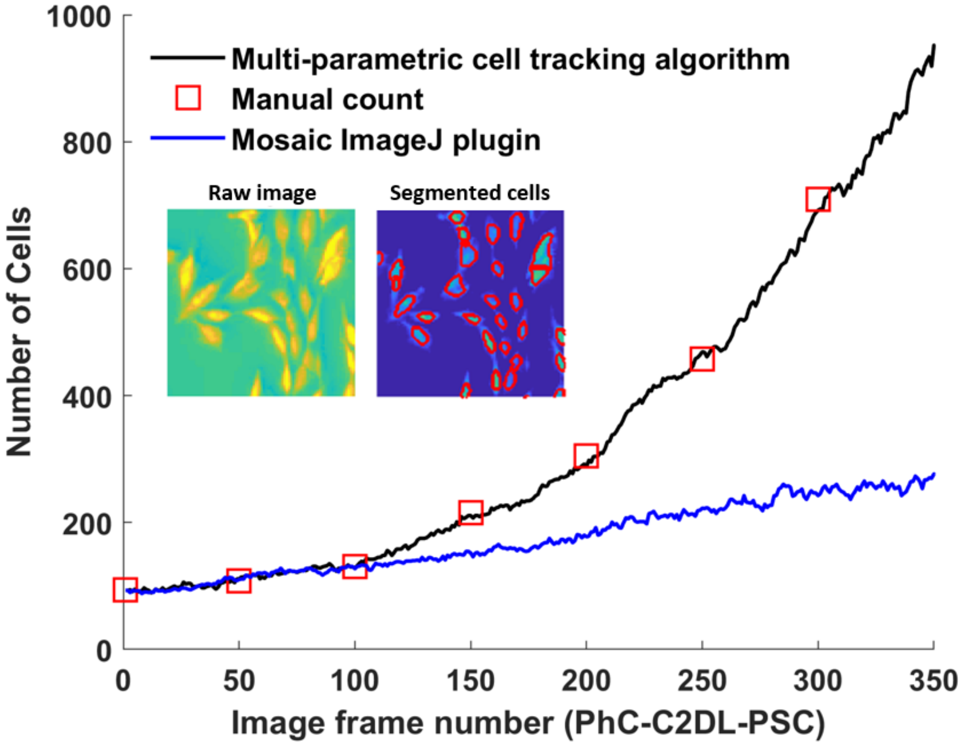

Figure S1. Number of cells counted from the published cell migration images using our cell tracking algorithm, Mosaic ImageJ plugin, and manual counting.

Microfluidic chamber

The µ–Slide Chemotaxis chamber is a well characterized commercial tool for the investigation of chemotactic migration of adherent mammalian cells (such as cancer and endothelial cells) within 3D matrices [36, 37]. While there are a number of well-established chamber designs available to study cell migration and chemotaxis [38-41], the µ–Slide Chemotaxis chamber was chosen to utilize highly standardized and minimally-sized cell culture areas under a sustained chemotactic gradient for multiple days. Subsequently, user fabrication and experimental variability is minimized while allowing a multiplex image acquisition with multiple chambers at once. Figure S3 illustrates the microfluidic chamber design.

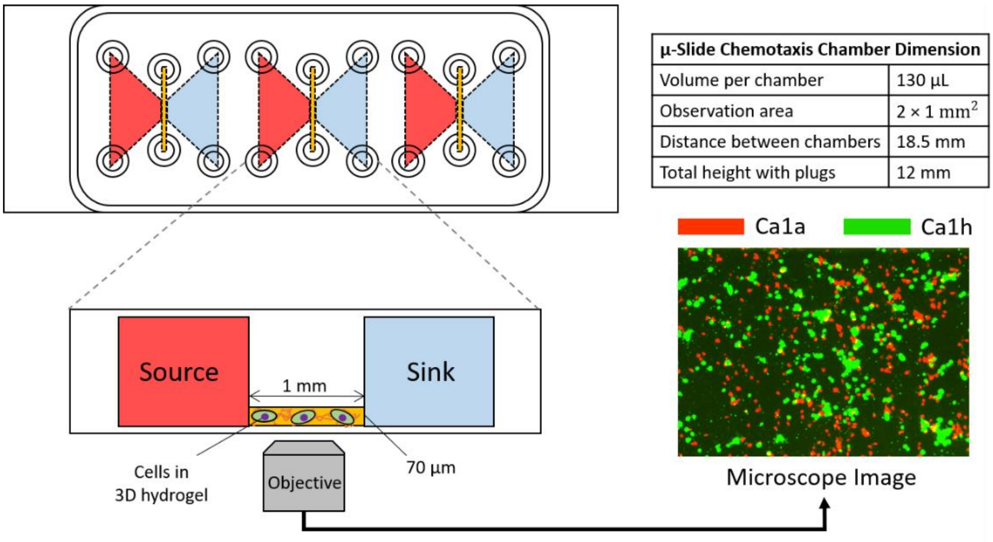

µ-Slide Chemotaxis Chamber Dimension	
Volume per chamber	130 µL
Observation area	2×1 mm^2
Distance between chambers	18.5 mm
Total height with plugs	12 mm

Figure S2. Ca1a and Ca1h cells in mono-culture and co-culture within µ–Slide Chemotaxis for measuring proliferation, velocity, and trajectory.

© 2020 by the authors. Submitted for possible open access publication under the terms and conditions of the Creative Commons Attribution (CC BY) license (http://creativecommons.org/licenses/by/4.0/).
